# Supplementary material for: Leveraging heterogeneity across multiple datasets increases cell-mixture deconvolution accuracy and reduces biological and technical biases
Source: Nat Commun. 2018 Nov 9;9:4735. doi: 10.1038/s41467-018-07242-6 (PMC6226523; doi:10.1038/s41467-018-07242-6)
Supplement: Supplementary file 1 — Supplementary Information [file 41467_2018_7242_MOESM1_ESM.pdf]

Leveraging heterogeneity across multiple datasets increases cell-mixture deconvolution accuracy and reduces biological and technical biases

Supplementary Information

Supplementary Figure 1

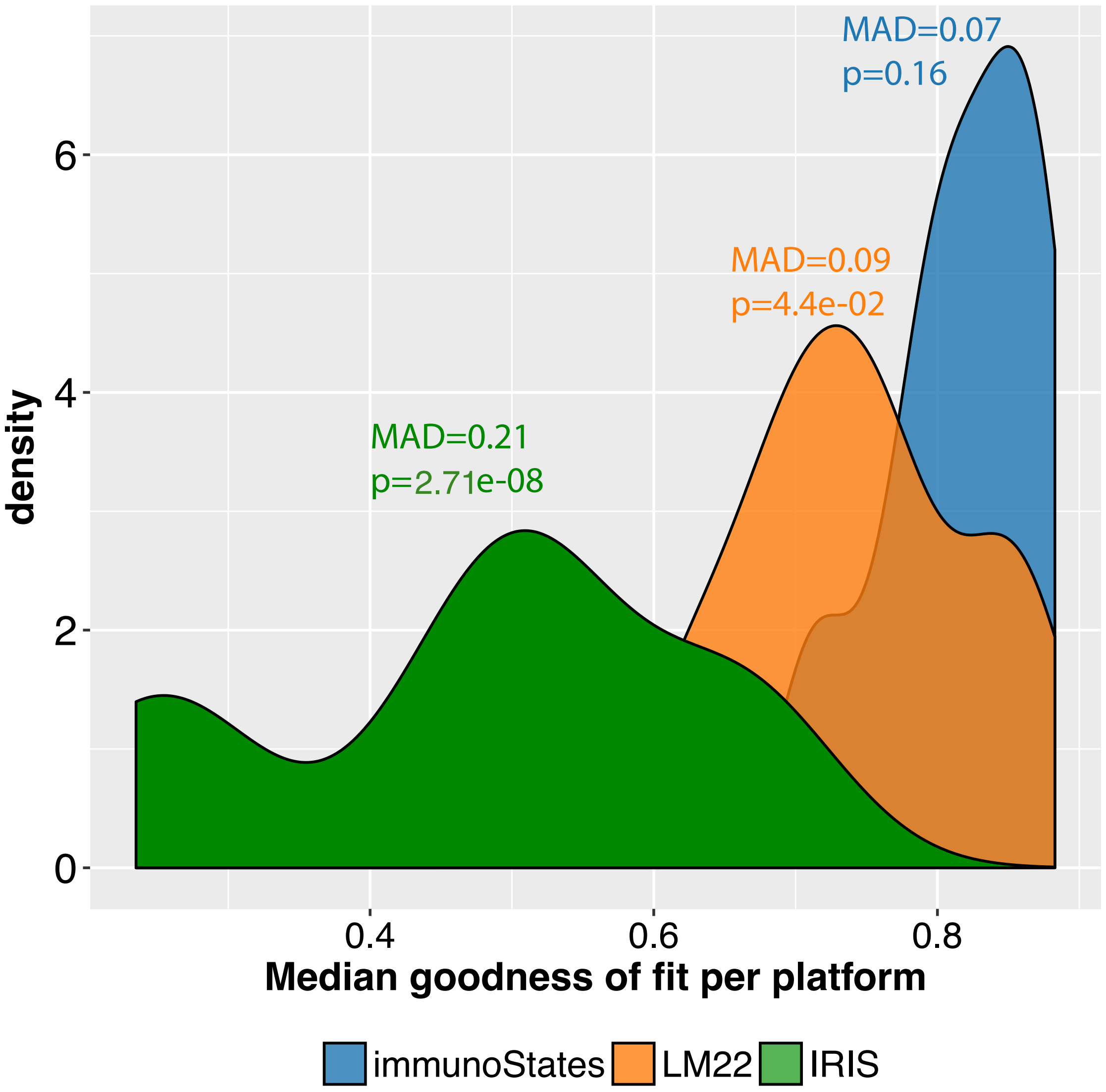

Platform bias in cell mixture deconvolution. Density plots representing the distribution of median goodness of fit for each platform across all methods grouped by different matrices (represented by fill color). Significance of platform bias is computed by estimating the Median Absolute Distance (MAD) of each distribution and comparing it to a null distribution that assumes no technical variation between samples.

Supplementary Figure 2

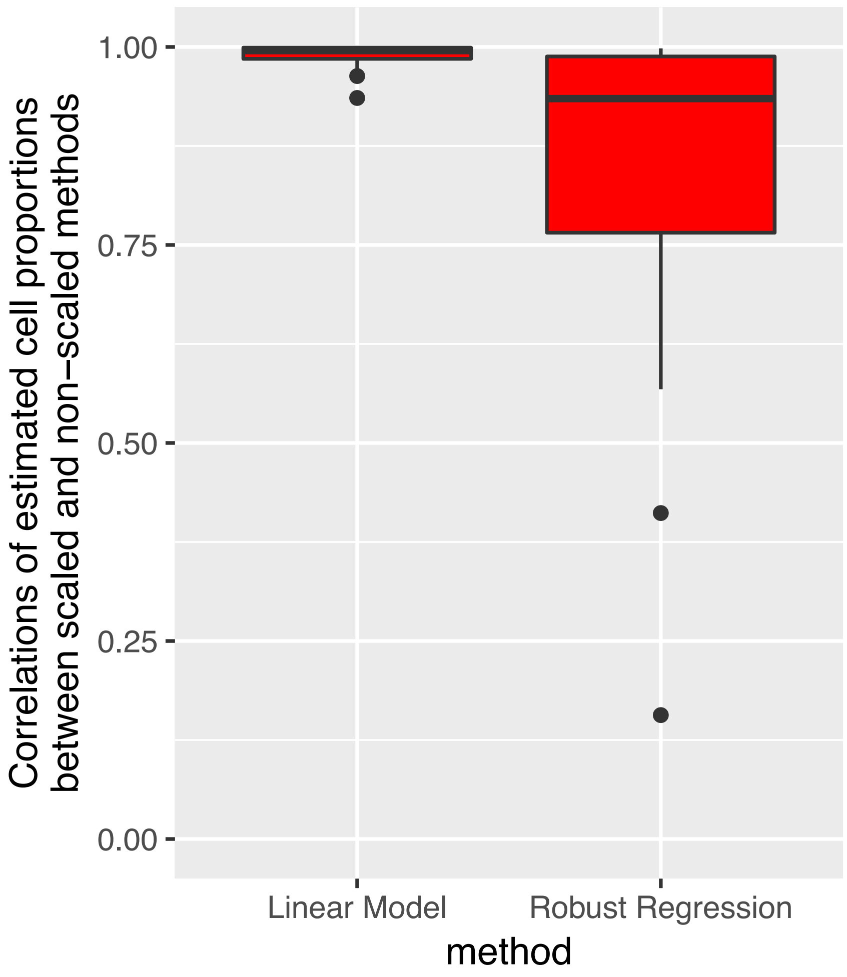

Effect of rescaling expression data to deconvolution. Boxplot displaying sample-level correlation between cell proportions estimated deconvolution with and without rescaling by either Linear Model (r = 0.989 +/- 0.002) or Robust Regression (r = 0.843 +/- 0.034) across multiple samples and basis matrices. Center lines correspond to the median value of each box and the lower and upper bounds of each box correspond to their first and the third quartiles, respectively.

Supplementary Figure 3

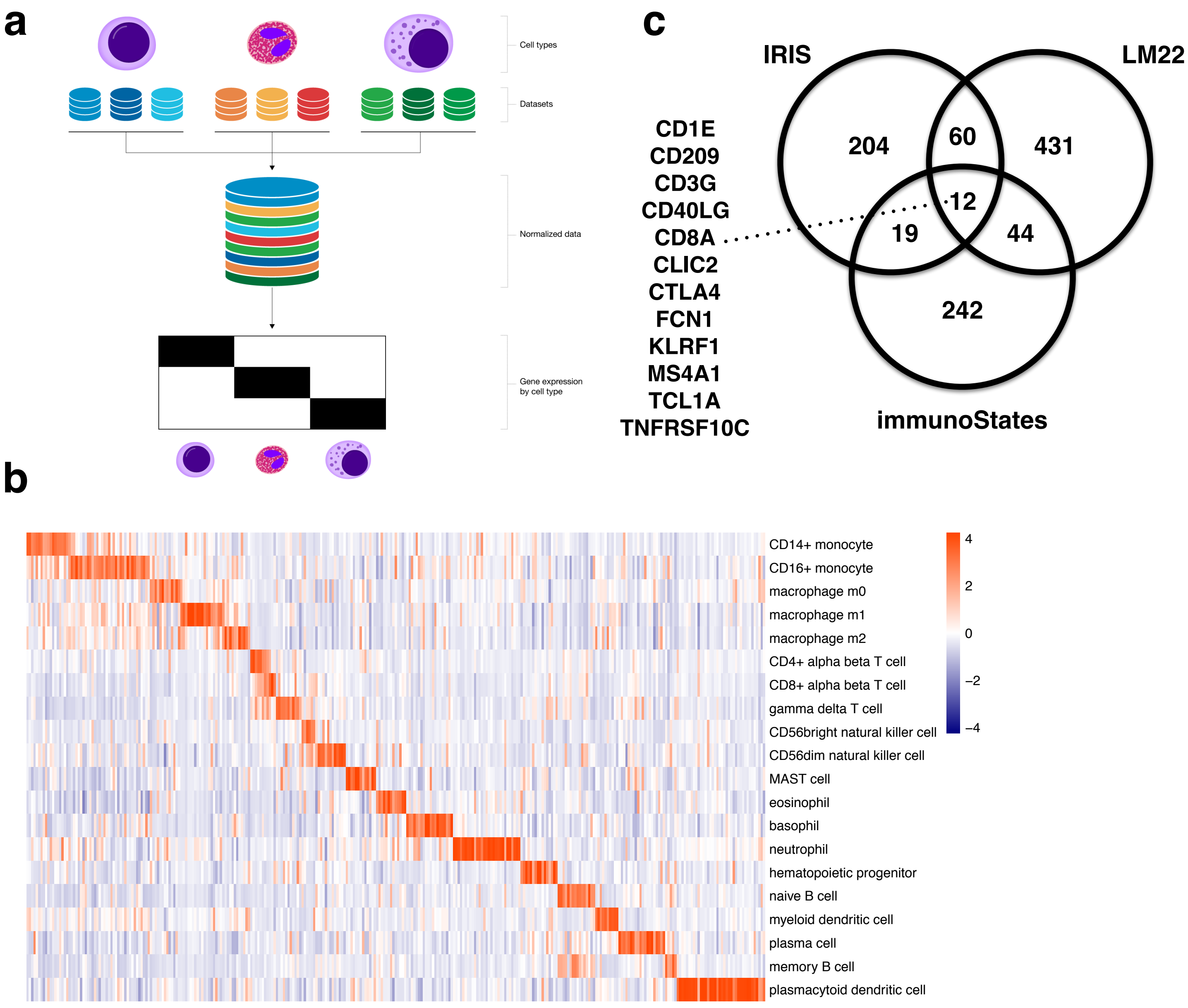

Creation of the immunoStates matrix. **(a)** Flow-chart describing the steps for the creation of the immunoStates expression matrix. **(b)** Heatmap showing expression of the immunoStates signature genes in target cell types. Genes expression values are displayed as z-scores per gene across all cell types. **(c)** Venn-diagram depicting the overlap between gene-sets between each basis matrix. Genes overlapping across all three matrices are listed on the left side of the diagram.

Supplementary Figure 4

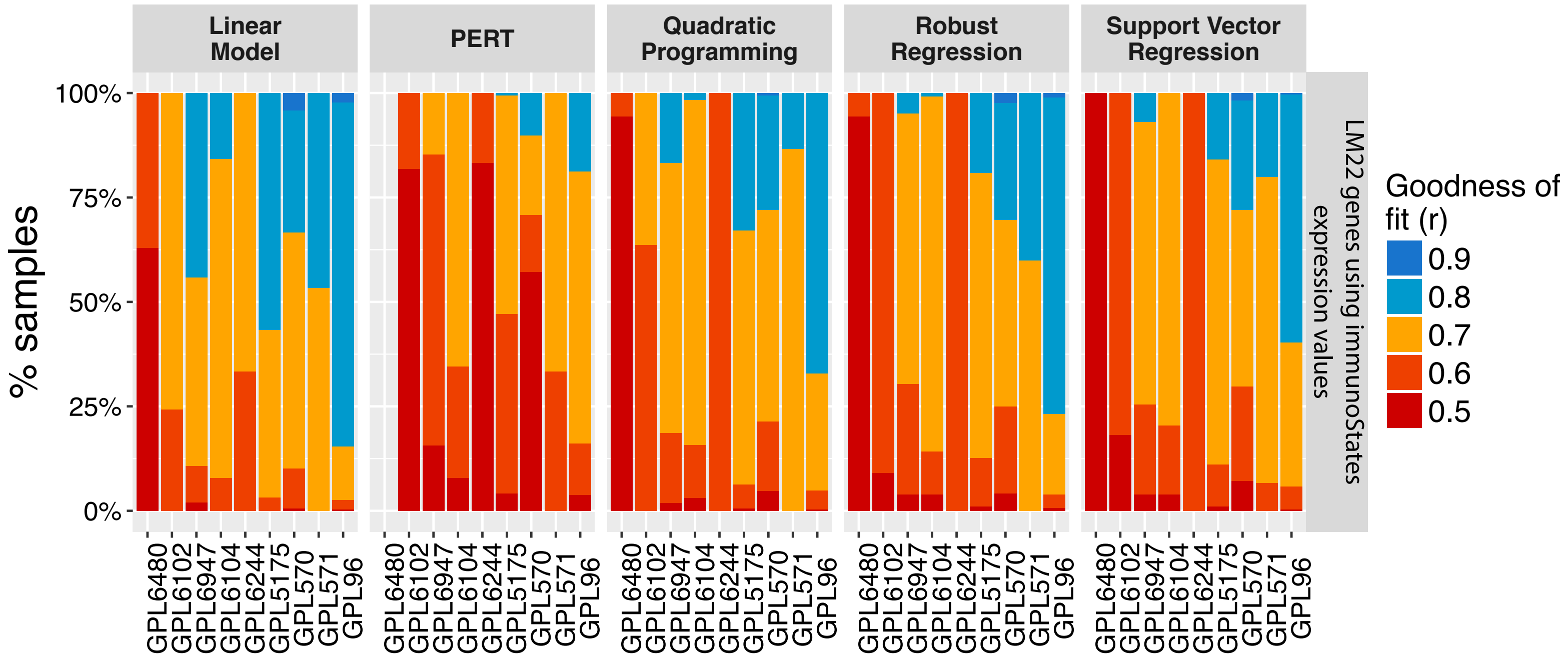

Increasing the amount of data to estimate expression values for genes in a basis matrix does not improve deconvolution accuracy. We used the datasets used to create immunoStates to calculate expression values for each gene in LM22, and deconvolved the technical bias evaluation cohort. We found increasing the amount of data to estimate expression value of a gene in a basis matrix did not increase accuracy.

Supplementary Figure 5

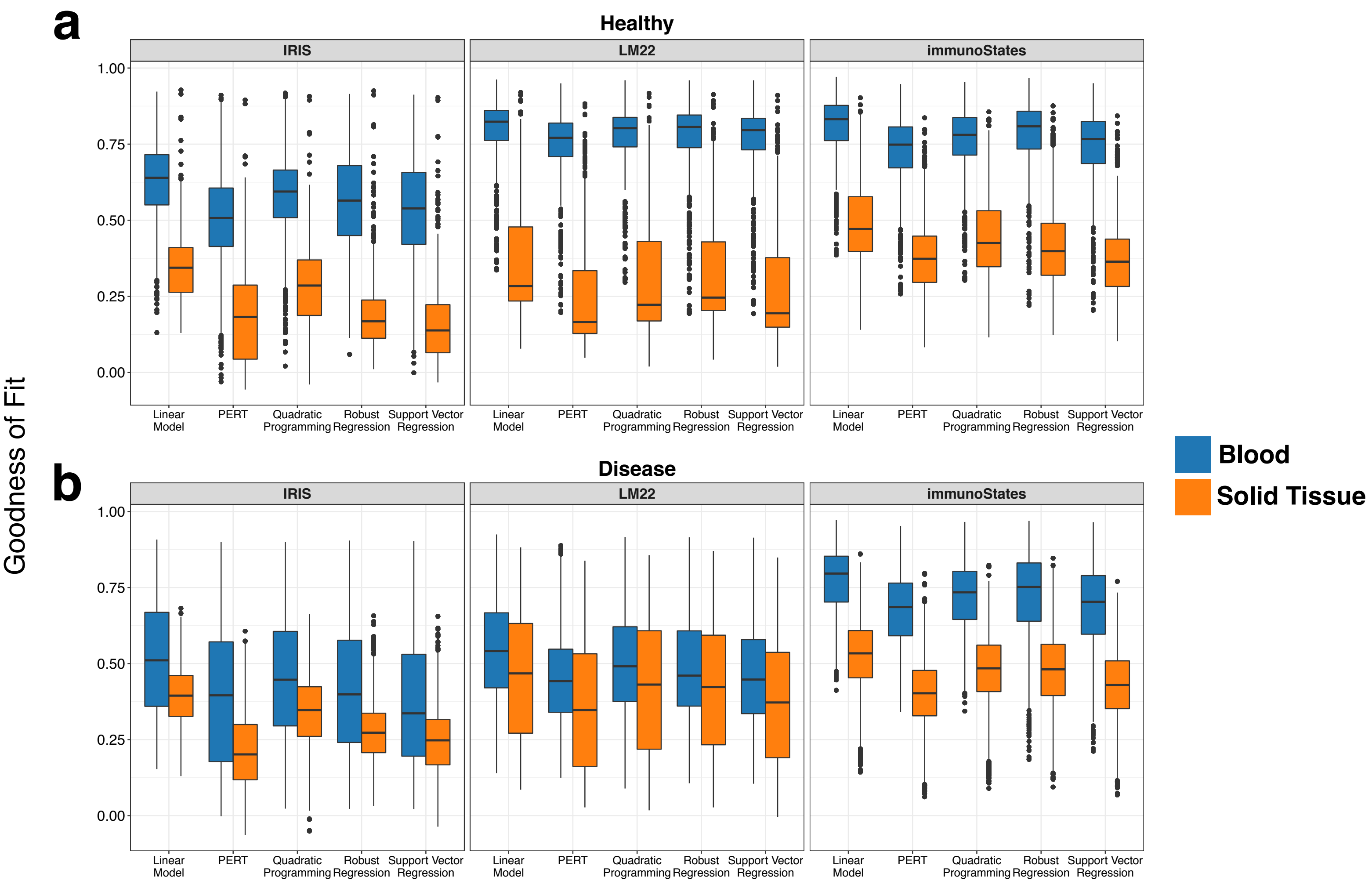

Goodness of fit in healthy and diseased samples. (a) Boxplots indicating goodness of fit scores (y-axis) for blood-derived and tissue-derived samples in healthy donors (1383 samples) across multiple deconvolution methods (x-axis) for IRIS, LM22, and immunoStates. Center lines correspond to the median value of each box and the lower and upper bounds of each box correspond to their first and the third quartiles, respectively. (b) Same as in (a) but in disease samples (2684 samples).

Supplementary Figure 6

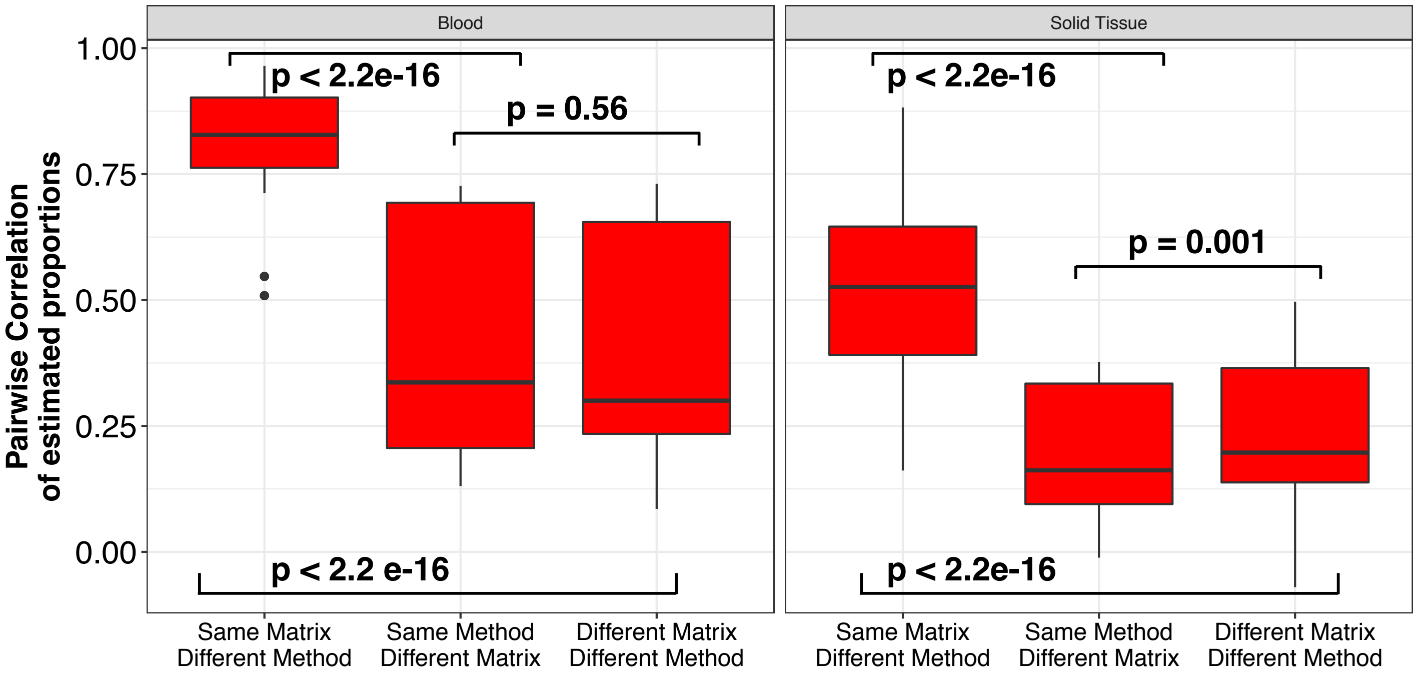

Deconvolution concordance by matrix and method across blood and solid tissue. Boxplots representing the distribution of pairwise correlation coefficients between estimated proportions for all matrices and deconvolution methods. Comparisons were divided in (1) pairs with the same signature matrix but run with different methods, (2) pairs with different signature matrices but run using the same method, and (3) pairs where both matrix and method were different. Significance analysis was performed using the Wilcoxon's paired rank sum test. Results are shown for samples containing blood cells or solid tissue biopsy from Lukk *et al* 2010.

Supplementary Figure 7

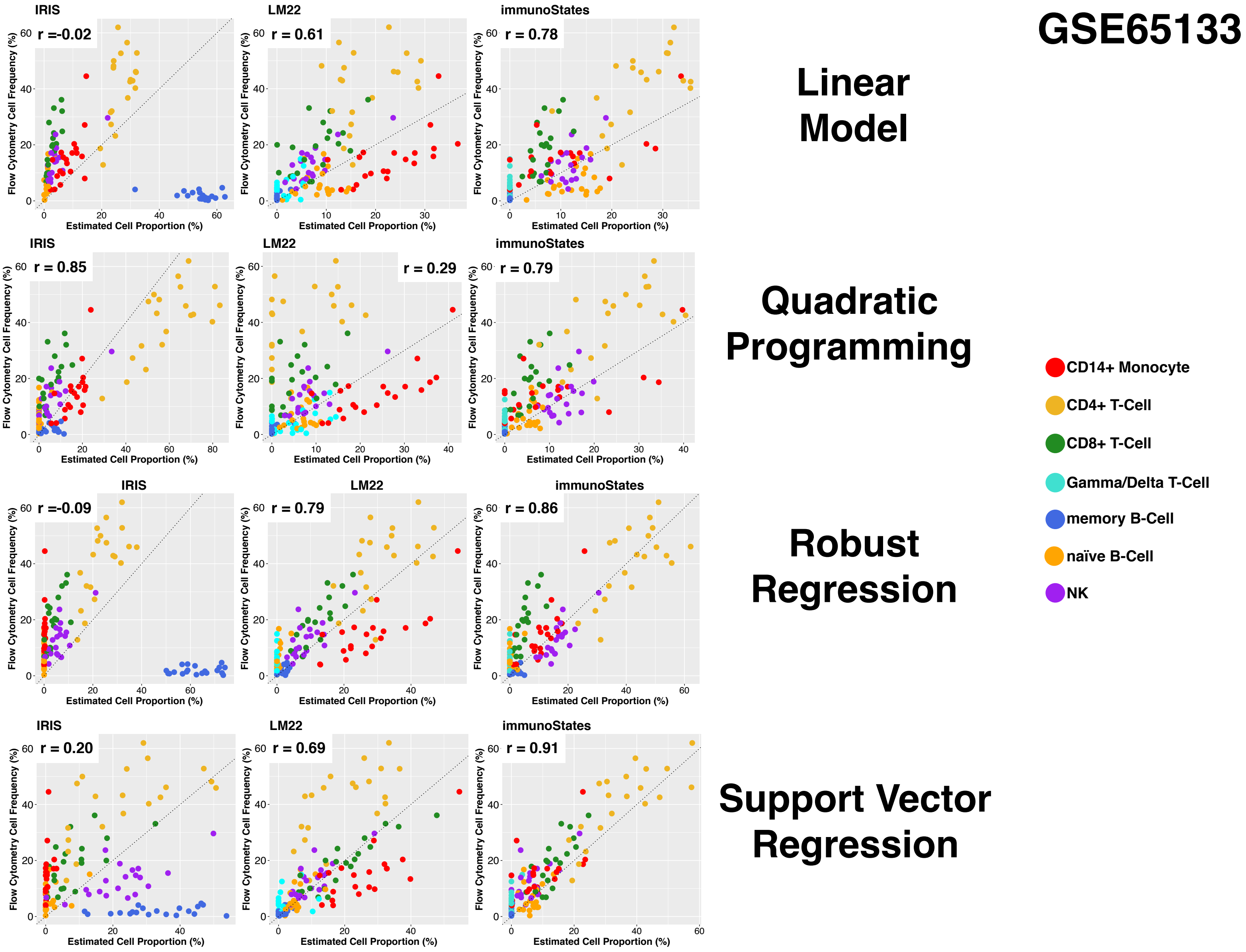

Supplementary Figure 8

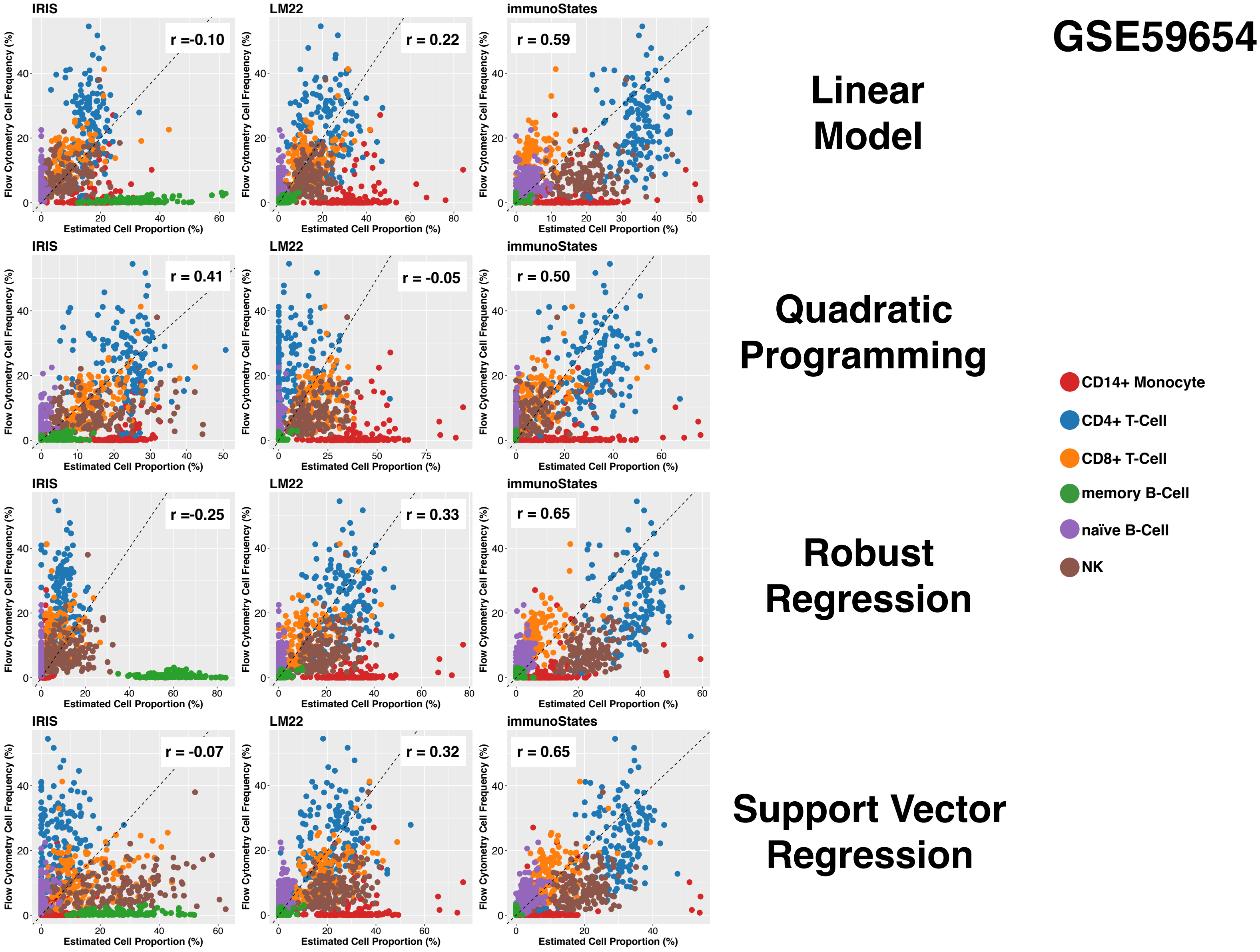

Supplementary Figure 9

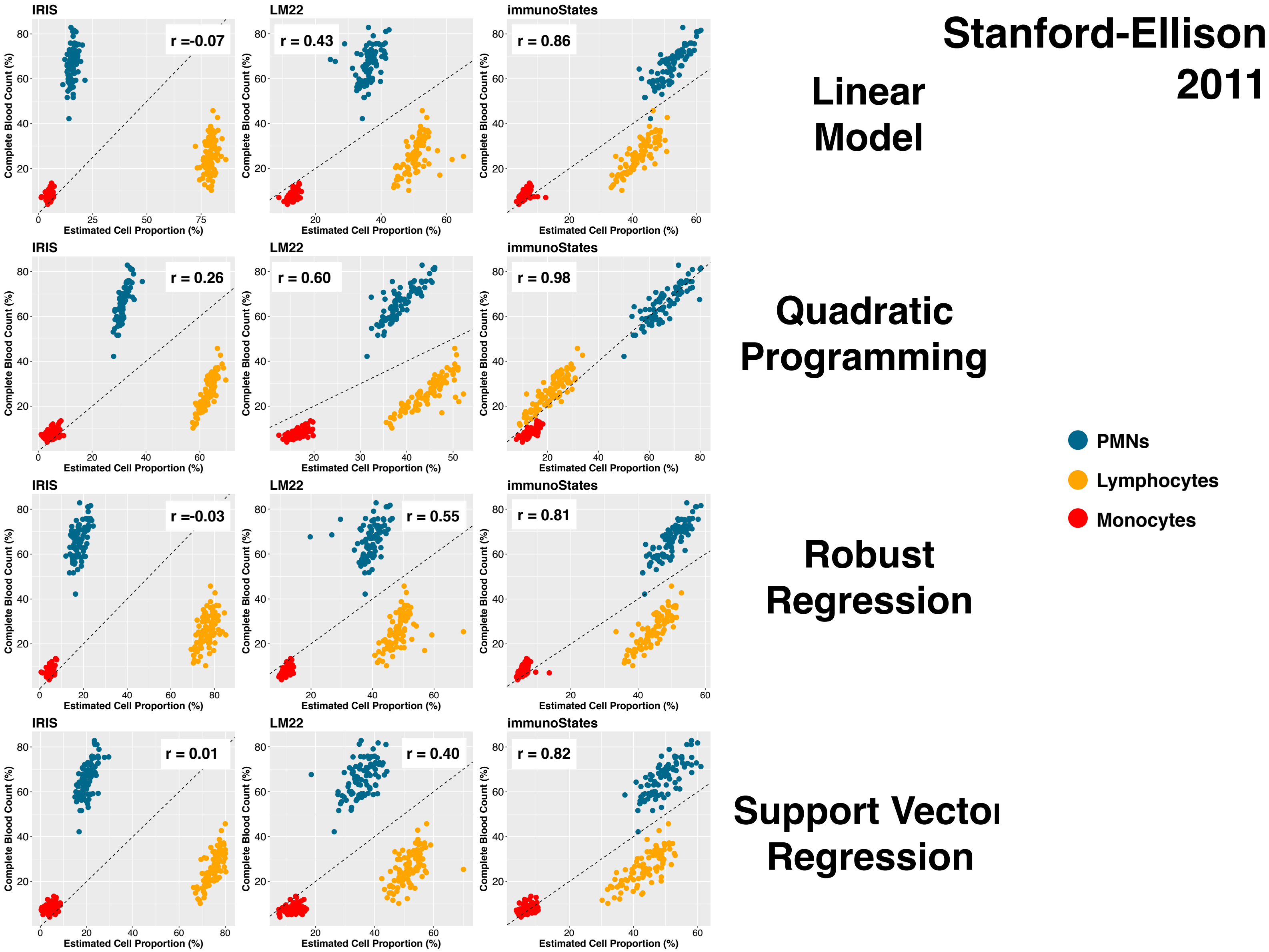

Supplementary Figure 10

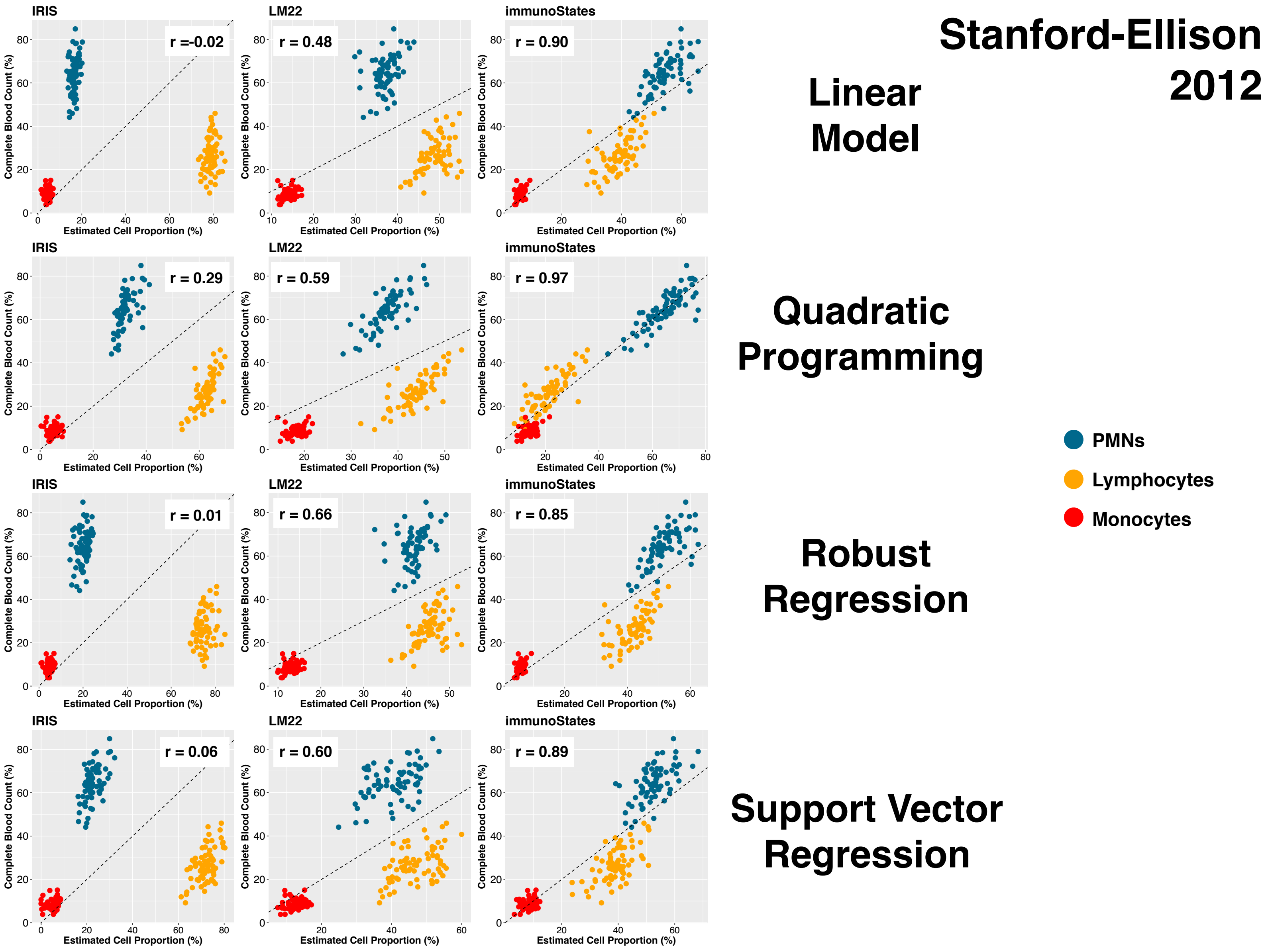

Supplementary Figure 11

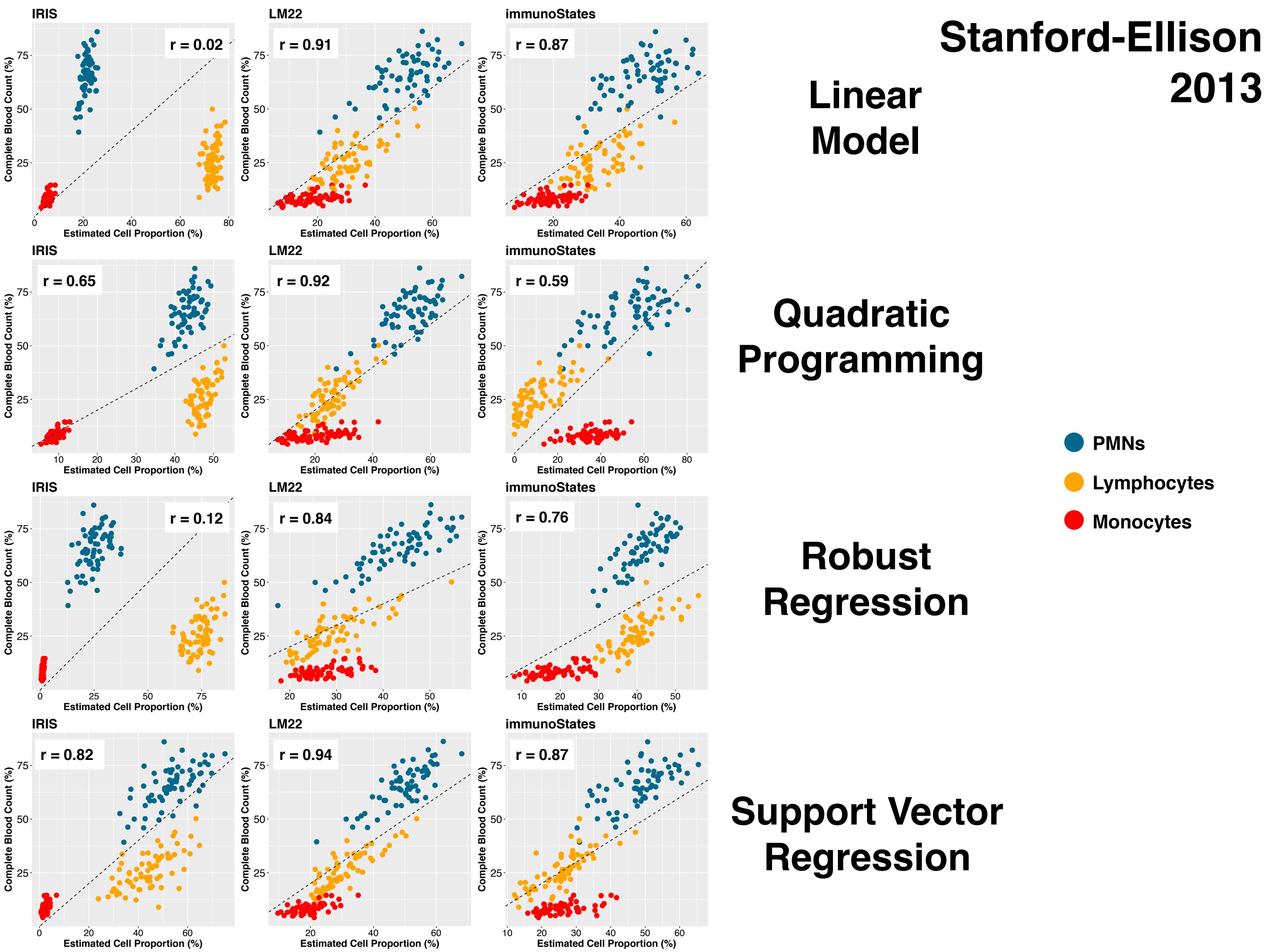

Supplementary Figure 12

GSE65133
PERT

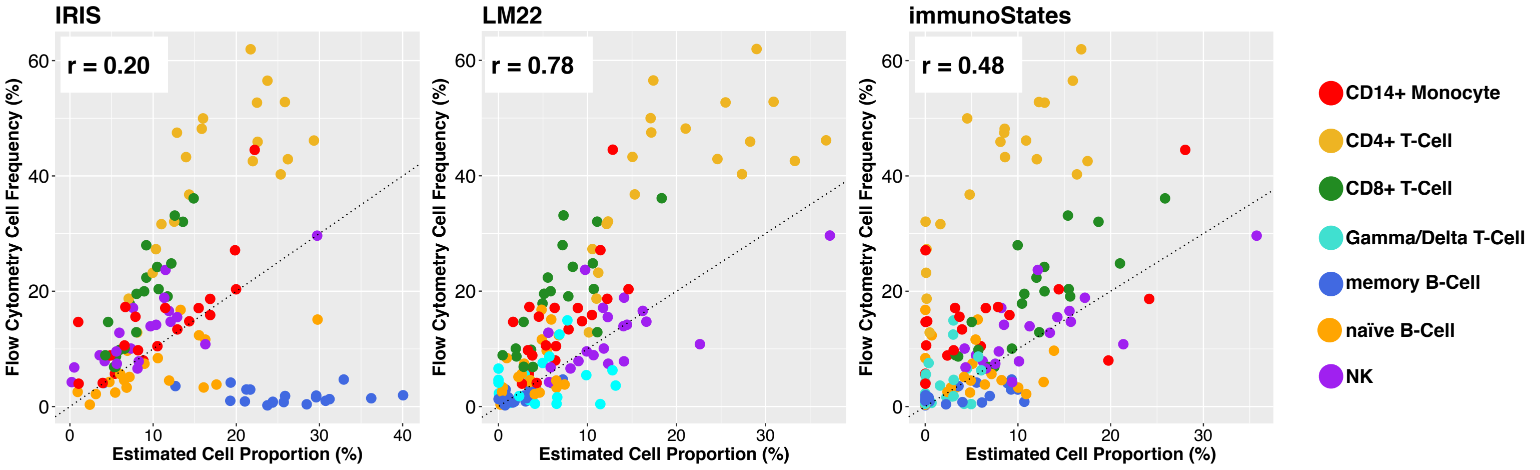

Supplementary Figure 13

GSE59654
PERT

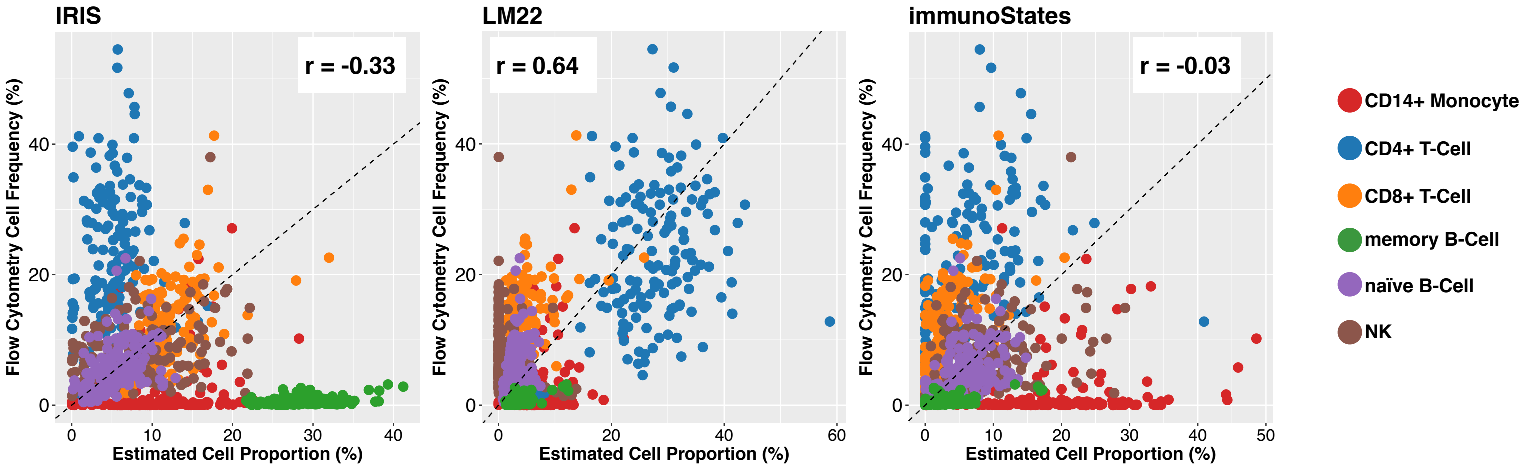

Supplementary Figure 14

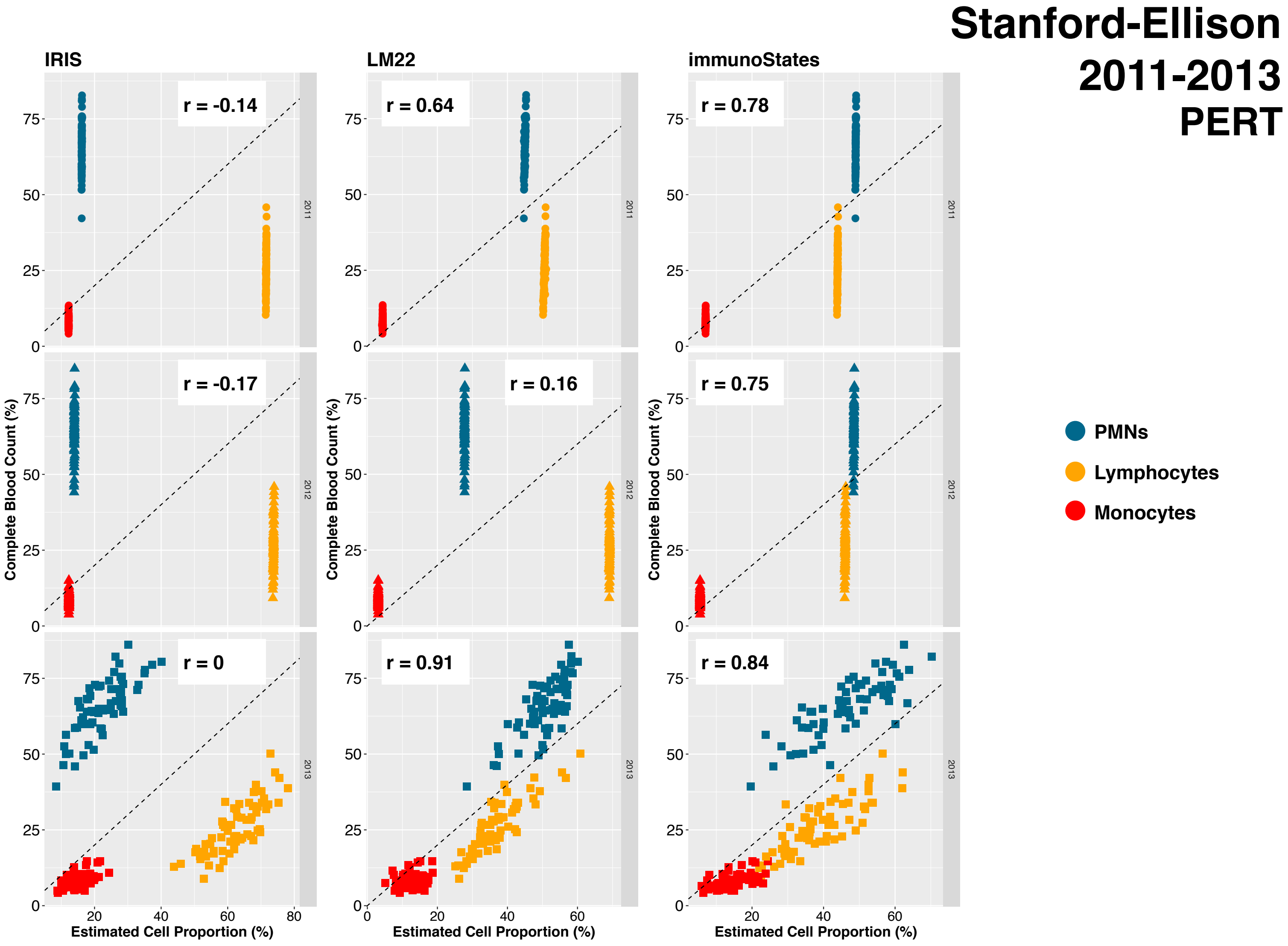

Correlation plots between measured and estimated proportions. (7) Correlation plots of estimated (x-axis) and measured cell proportions (y-axis) for each method and matrix combination for samples in GSE65133. Correlation is measured by Pearson's correlation coefficient. **(8)** Same as in **(7)** for GSE59654. **(9,10,11)** Same as in **(7)** for Stanford-Ellison 2011, 2012, and 2013 sample cohorts respectively. **(12,13,14)** Same as in previous figures but using the PERT method.

Supplementary Figure 15

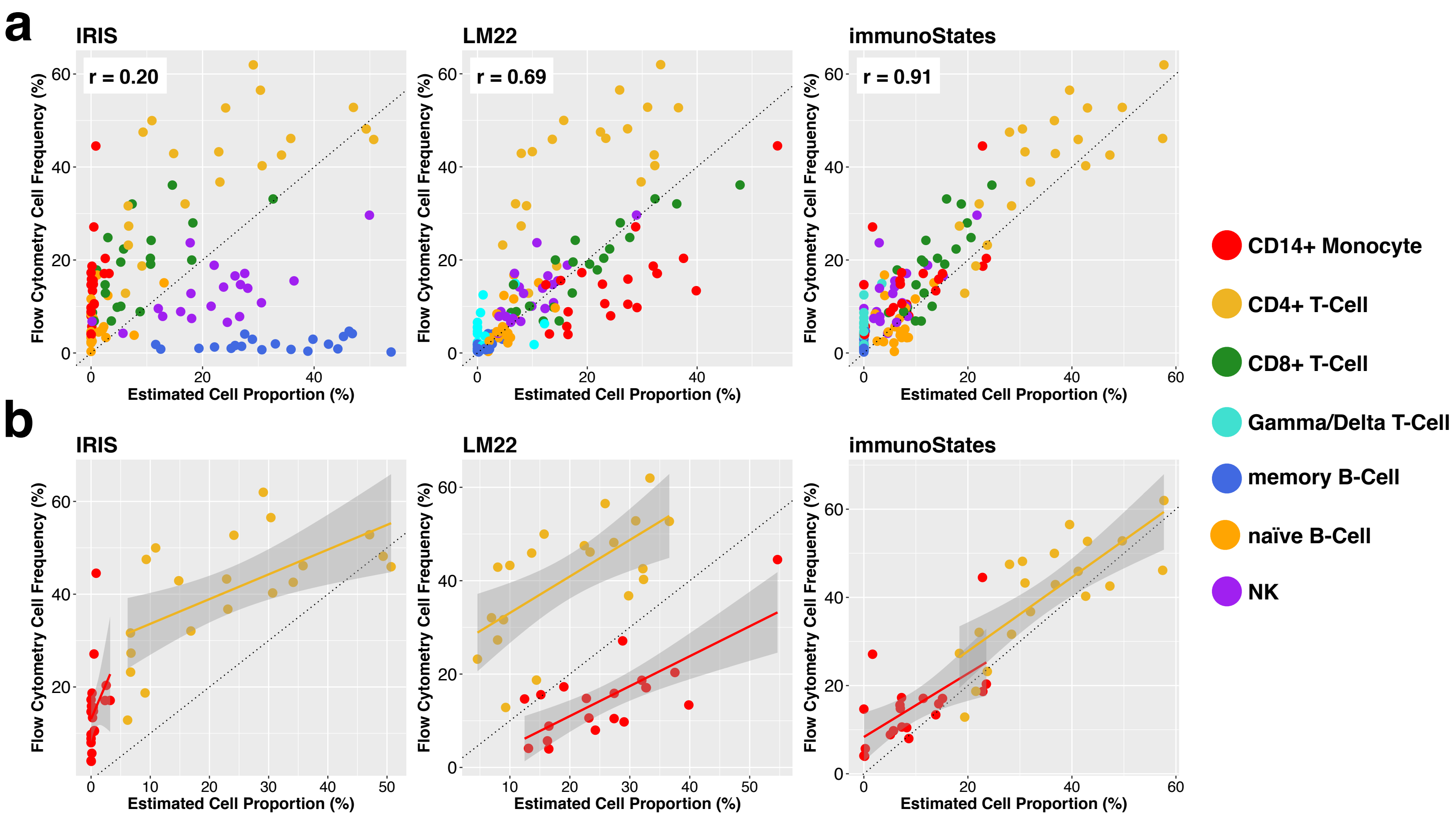

Example of systematic under- and over-estimation of cell proportions in cell mixture deconvolution. (a) Correlation plots of estimated (x-axis) and measured cell proportions (y-axis) in GSE65133 for all matrices using Support Vector Regression. **(b)** Highlighted CD4+ T-cell (yellow) and Monocyte (red) cell proportion estimates against measured values. Solid lines represent the best fit with a linear model, whereas the dashed line represent the 45-degree diagonal.

Supplementary Figure 16

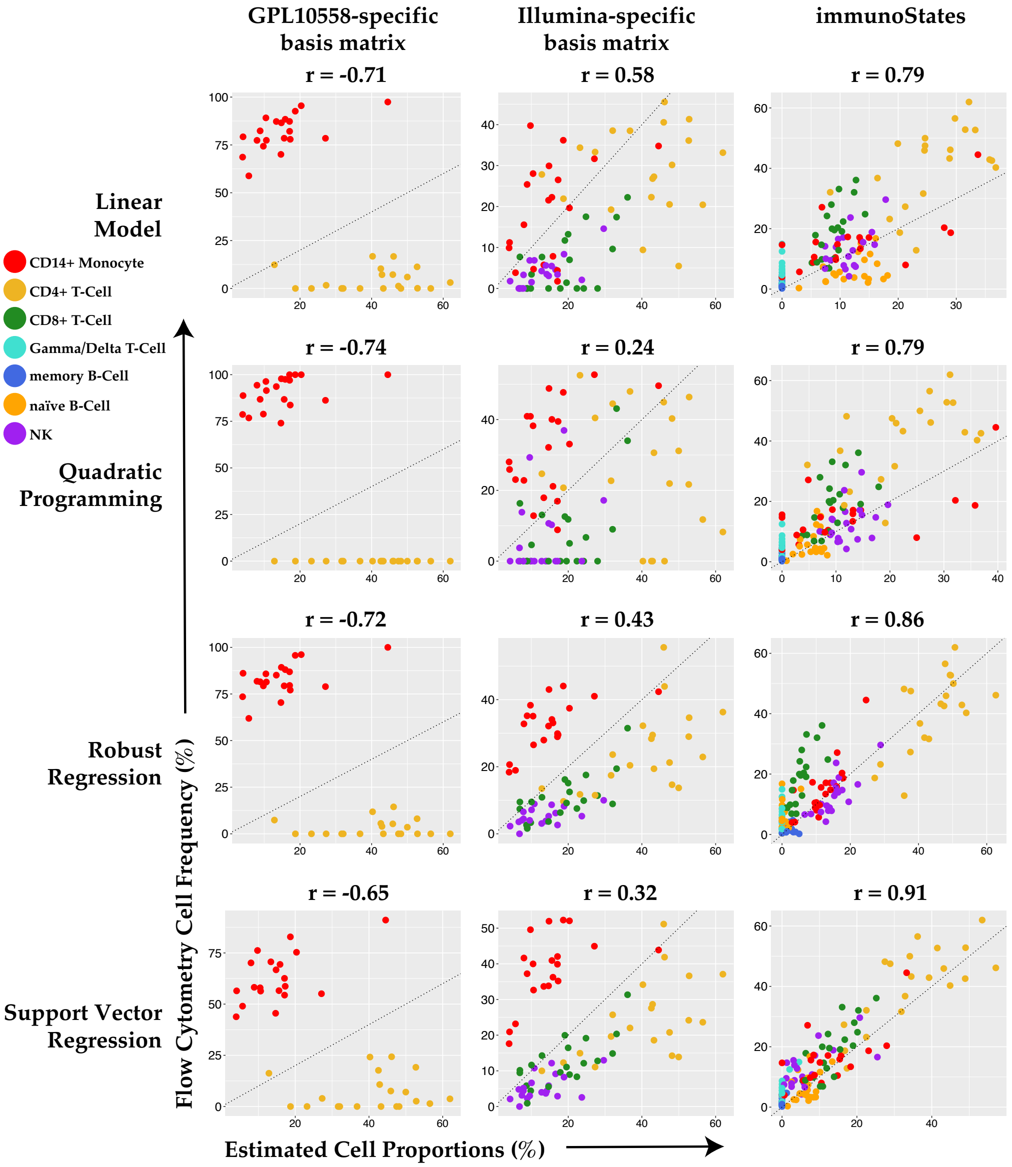

Comparison of Illumina-specific basis matrices with immunoStates. GPL10558-specific basis matrix, used in the first column, was created using only sorted immune cell gene expression profiles using GPL10558; Illumina-specific basis matrix, used in the second column, was created using sorted immune cell gene expression profiles using any Illumina microarrays; the third column used immunoStates for deconvolution. Irrespective of the method used, estimated cell proportions using immunoStates has higher correlation than both Illumina- specific basis matrices when deconvoluting GSE65133 that was generated using Illumina-based microarrays, GPL10558.

Supplementary Table 1

Datasets used to measure platform bias			
GSE	***GPL***	***Brand***	***Samples***
GSE38958	GPL5175	Affymetrix	115
GSE37912	GPL5175	Affymetrix	74
GSE21942	GPL570	Affymetrix	29
GSE19314	GPL570	Affymetrix	58
GSE22356	GPL570	Affymetrix	30
GSE55098	GPL570	Affymetrix	22
GSE17114	GPL570	Affymetrix	29
GSE17393	GPL571	Affymetrix	15
GSE23832	GPL6244	Affymetrix	12
GSE14577	GPL96	Affymetrix	15
GSE11907	GPL96	Affymetrix	122
GSE11909	GPL96	Affymetrix	115
GSE9006	GPL96	Affymetrix	105
GSE3365	GPL96	Affymetrix	68
GSE15573	GPL6102	Illumina	33
GSE18885	GPL6104	Illumina	127
GSE33463	GPL6947	Illumina	102
