# Supplementary material for: Leveraging heterogeneity across multiple datasets increases cell-mixture deconvolution accuracy and reduces biological and technical biases
Source: Nat Commun. 2018 Nov 9;9:4735. doi: 10.1038/s41467-018-07242-6 (PMC6226523; doi:10.1038/s41467-018-07242-6)
Supplement: Supplementary file 3 — Description of Additional Supplementary Files [file 41467_2018_7242_MOESM3_ESM.pdf]

Description of Additional Supplementary Files

File Name: Supplementary Data 1
Description: Annotation table for gene expression datasets used to create immunoStates

File Name: Supplementary Data 2
Description: The immunoStates basis matrix
